# Motivation and satisfaction among community health workers administering rapid diagnostic tests for malaria in Western Kenya

**DOI:** 10.7189/jogh.08.010401

**Published:** 2018-06

**Authors:** Laura K Winn, Adriane Lesser, Diana Menya, Joy N Baumgartner, Joseph Kipkoech Kirui, Indrani Saran, Wendy Prudhomme-O’Meara

**Affiliations:** 1 Duke University, Durham, North Carolina, USA; 2 Duke University, Duke Global Health Institute, Durham, North Carolina, USA; 3 Moi University, School of Public Health, Eldoret, Kenya; 4 Academic Model Providing Access to Healthcare (AMPATH), Eldoret, Kenya

## Abstract

**Background:**

The continued success of community case management (CCM) programs in low-resource settings depends on the ability of these programs to retain the community health workers (CHWs), many of whom are volunteers, and maintain their high-quality performance. This study aims to identify factors related to the motivation and satisfaction of CHWs working in a malaria CCM program in two sub-counties in Western Kenya.

**Methods:**

We interviewed 70 CHWs who were trained to administer malaria rapid diagnostic tests as part of a broader study evaluating a malaria CCM program. We identified factors related to CHWs’ motivation and their satisfaction with participation in the program, as well as the feasibility of program scale-up. We used principal components analysis to develop an overall CHW satisfaction score and assessed associations between this score and individual CHW characteristics as well as their experiences in the program.

**Results:**

The majority of CHWs reported that they were motivated to perform their role in this malaria CCM program by a personal desire to help their community (69%). The most common challenge CHWs reported was a lack of community understanding about malaria diagnostic testing and CHWs’ role in the program (39%). Most CHWs (89%) reported that their involvement in the diagnostic testing intervention had either a neutral or a net positive effect on their other CHW activities, including improving skills applicable to other tasks. CHWs who said they strongly agreed with the statement that their work with the malaria program was appreciated by the community had a 0.76 standard deviation (SD) increase in their overall satisfaction score (95% confidence interval CI = 0.10-1.24, *P* = 0.03). Almost all CHWs (99%) strongly agreed that they wanted to continue their role in the malaria program.

**Conclusions:**

Overall, CHWs reported high satisfaction with their role in community-based malaria diagnosis, though they faced challenges primarily related to community understanding and appreciation of the services they provided. CHWs’ perceptions that the malaria program generally did not interfere with their other activities is encouraging for the sustainability and scale-up of similar CHW programs.

Community Health Workers (CHWs) are a broad category of lay people who typically work outside of formal health facilities to provide health services for members of the community. CHWs work across many contexts, but are especially crucial in providing health services in areas where there are few health workers, limited access to health care, and/or large disparities in health outcomes. CHWs do not typically receive formal professional training or a specialized degree, but are selected and trained to perform specific health tasks. For the most part, CHWs are volunteers [1]. CHWs have been reported to provide care that many community members perceive as comparable to care provided in health facilities [2-7].

Although they have far-reaching roles in health education and health promotion, a handful of more clinical interventions have been implemented through community health workers including Community Case Management (CCM) programs, which target prompt diagnosis and treatment in populations with inadequate access to formal health facilities and services [8,9]. The World Health Organization’s recent launch of the Rapid Access Expansion Programme to scale-up integrated CCM (iCCM) demonstrates increasing global interest and support for expanding CCM programs to address childhood illnesses [10,11].

Since CHWs play a key role in providing health services, and in many cases they work on a voluntary basis, their personal satisfaction and motivation are central to their continued involvement. As CHWs are increasingly being deployed in community health programs, a better understanding of CHW motivation and satisfaction is needed not only to promote their well-being, but also to improve their performance and retention. Better performance enhances the quality of CCM programs, and improved retention reduces the need to continually recruit and train new CHWs, thus promoting the sustainability and scalability of programs that rely on CHW services [2,12-17].

To understand the motivational factors of CHWs for this study, we adapted a four-level organizational system of motivators from Greenspan et al. [18] to categorize existing evidence on CHW motivators at the individual level, family level, community level, and organizational level. In addition, this framework helped us in our data interpretation. At the individual level, research points to altruism, a desire to serve their community, personal relationships, and personal opportunities to learn new skills as powerful intrinsic motivators for CHWs [19-23]. They take pride in their work and are encouraged by the competence and autonomy they have in their roles [12,21]. At the family level, the moral and material support CHWs receive enables them to work effectively in their roles [19,21].

Equally important are motivators at the community level including appreciation, recognition and respect for CHWs’ contribution to their communities [13,19-22,24,25]. Many communities even provide monetary, material, and work-related support [21,24]. Lastly, organizational-level factors that contribute to CHW motivation include effective supervision, financial compensation, and non-financial material incentives (bicycles, T-shirts, badges, phones, job aides, supplies) [13,19,21-23]. CHWs also appreciate the skills, knowledge, and formal recognition they gain from training [19,21-23,26-28]. Formal linkage to the health system and recognition of their role within that system are also frequently cited as factors in maintaining CHW morale [12,13,24]. On the other hand, when CHWs experience challenges such as high patient load, limited supplies, limited knowledge and supervision, lack of compensation, and unrealistic expectations from community members, this puts a strain on their altruistic motivations for doing their work [20,28].

While much research has been done to assess CHW motivation, there is less evidence on how CHWs perceive their role and their motivation and satisfaction with their work when they provide more clinical services such as diagnostic testing as part of CCM programs [5,29]. Given the important role of individual satisfaction and motivation in ensuring high performance and quality of services, we sought to measure factors related to CHW satisfaction and motivation in this malaria CCM program and the feasibility of scale-up of malaria CCM programs that rely on a volunteer community health workforce.

## METHODS

### Study context and population

This study was part of a stratified cluster-randomized controlled trial that aims to evaluate the public health impact of community-based malaria diagnostic testing. The study was conducted in 32 community units in two sub-counties in western Kenya (Bungoma East and Kiminini). A community unit (CU) consists of approximately 1000 households (5000 people), 20 volunteer CHWs and one Community Health Extension Worker (CHEW) government employee who supervises the CHWs [30]. In order to be eligible to participate in the study, a CU had to have an existing system of trained CHWs. The 32 eligible CUs in the area were randomly assigned to either the intervention or control arms (with 16 CUs per arm).

In the intervention CUs, CHWs were trained to perform malaria rapid diagnostic tests (RDTs) using a validated 3-day curriculum (based on the Kenya Ministry of Health curriculum) in conjunction with practical, skills-oriented sessions. The study team provided CHWs with the necessary supplies to conduct the RDT testing monthly to ensure that no CHW was ever out of supply of RDTs [30]. The RDT used was the CareStart HRP2, which detects malaria infections caused by *P. falciparum,* which is responsible for the majority of malaria cases in Kenya [31]. CareStart HRP2 RDTs have a sensitivity of 98.7% and a specificity of 92.5-95% [32].

Over the entire intervention period, each CHW performed an average of 5.7 RDTs/mo. This number varied across community units from between 1.5 tests/mo and 16 tests/mo. Data collected from a sample of households in the area at the baseline of this study shows that only approximately 22% (443/2020) had previous experience with RDTs (unpublished data). CHWs in this program do not dispense anti-malarial drugs, but they provide vouchers for malaria-positive clients to receive highly subsidized first-line anti-malarial drugs (artemether-lumefantrine) from local drug shops [30]. In the control arm, CHWs continued to provide their usual services, which include health education, basic treatments (such as first-aid) and referrals to health care facilities [33]. The randomized controlled trial is described in more detail in a previously published paper [30].

In Kenya, CHWs work on a volunteer basis while Community Health Extension Workers (CHEWs) are employees of Kenya’s Ministry of Health (MOH). CHEWs are required to have a certification in either nursing or public health, and their role is to supervise the CHWs. Though it is not required for the CHWs to have a degree to be selected for their role, the MOH recommends that CHWs be able to read and write. CHWs’ activities outside of the malaria program varied, but typically included health education on water, sanitation, and disease prevention, referrals of sick clients from the community to the formal health sector, occasional vaccine campaigns, and other short-term activities [33]. The CHWs performing RDTs in this malaria study were not paid but received reimbursement for transport to attend monthly supervision meetings with study staff (200-400 KSH/mo depending on distance to meeting place) and a small amount of mobile phone airtime (200 KSH/mo). In addition, as part of their participation in the program, every 6 months CHWs in the intervention areas were given a modest performance-based bonus as a group (defined by the community unit they serve). This group bonus was a maximum of 20 000 KSH every six months for the group of approximately 20 CHWs. Lastly, although not a direct incentive provided as part of the program, CHWs may expect that the skills and knowledge they gained as a result of participating in the malaria project will increase their ability to gain employment in other health programs [30]. In general, CHWs in the public sector in Kenya who are not engaged in externally funded activities do not receive transport reimbursement, mobile phone airtime, or a performance bonus.

We conducted interviews with a stratified random sample of 70 of the 273 (26%) CHWs participating in the study in the intervention areas between May and June 2016 (approximately nine to ten months after the start of the intervention). We stratified based on whether the study had assigned the CHW to use an electronic monitoring and evaluation device (a Deki reader) for a small portion of their tests [34,35]. 37% of our CHWs in the intervention group were assigned to utilize the Deki reader to assist in reading RDT results. We sampled CHWs using the “randbetween” function in Excel to randomly generate a number between one and ten thousand for each CHW. The CHWs were then sorted according to the randomly assigned number and 105 of the 273 (38%) were selected to participate in the study. We randomly interviewed CHWs within this list until our data reached saturation (n = 70 CHWs), a qualitative measure of the point at which additional interviews no longer contributed to variation in the data [36]. None of the CHWs who were selected to participate refused to be interviewed for this study. However, we were unable to contact two of the CHWs through their mobile phone number, and thus they were not interviewed for the study.

### Data

We designed the CHW survey through a collaborative and iterative process, including input from field staff closely involved in CHW training and day-to-day monitoring activities. We piloted the survey in both English and Kiswahili, after which it was revised to improve clarity and scope. The piloting was done with six CHWs who were part of the control arm of the malaria CCM program, and thus were not included in the final sample of CHWs interviewed for the survey. The survey collected demographic information about the CHWs, and asked questions about sources of CHW motivation, their satisfaction with their role in the program, challenges they have faced, and how the activities of the program have affected their other CHW and non-CHW responsibilities (see Appendix S1 in **Online Supplementary Socument(Online Supplementary Document)** for the questionnaire). We digitized and managed study data using REDCap electronic data capture tools hosted at Duke University. The survey had 57 questions and lasted between 45 minutes to 1 hour [37].

The semi-structured surveys were administered by trained interviewers who were not affiliated with the larger randomized controlled trial study team in order to encourage CHWs to be more comfortable providing feedback. The interviewers administered questionnaires verbally, in either Kiswahili or English according to the preference of the CHW. For most questions, participants could answer spontaneously (eg, their motivations, challenges, etc.), but we had a set of pre-categorized answers as well as the option “other” for recording responses that the interviewer believed did not fit into any of the previously coded answer choices. The interviewers asked the questions aloud without reading the responses to avoid leading the CHWs to give certain answers. Additionally, the interviewers asked the CHWs whether they had anything further to add before continuing to the next question to ensure that the CHWs had the time to give complete responses. In the analysis, we examined responses provided in the “other” category and, if appropriate, re-categorized them to one of the previously coded responses or, in some cases, created a new category (when at least 10% of responses fit into this new category). The majority of questions allowed more than one answer.

For some questions in the survey, the responses were based on a 5-point Likert-scale (“strongly agree”, “somewhat agree,” “neutral,” “somewhat disagree,” “strongly disagree”). To improve comprehension of this type of question, the interviewers provided the CHWs with a print of the five response options, in both English and Kiswahili, to refer to while the interviewer asked the question.

### Analysis

Means and proportions are presented to describe the demographic characteristics of the CHWs and their responses to questions on motivation and satisfaction, as well as their experiences participating in the malaria CCM program.

Polychoric principal component analysis (PCA) was utilized to create an overall CHW Satisfaction Score. Five questions were used to create the satisfaction score including 1) “Overall, I enjoy carrying out my role in this malaria project” (binary variable: strongly agree or otherwise), 2) “What do you like about participating in this project?” (count variable based on number of items mentioned when CHWs were asked what they liked and what they didn’t), 3) “What don't you like about participating in this project?” (count variable based on number of items mentioned), 4) “What motivates you to participate in this project?” (count variable based on number of items mentioned), and 5) “What are the main challenges you face in implementing this project?” (count variable based on number of items mentioned). The response to the first question on overall satisfaction was based on a 5-point Likert-Scale. However, since almost all respondents answered “strongly agree” to this question we dichotomized the response into “Strongly Agree” and “Other”.

The CHW satisfaction score was defined as the first principal component from the polychoric PCA. We used polychoric PCA to account for the fact that the variables used in creating the score may not be normally distributed [38]. We use univariate linear regressions to individually assess the association between the CHW satisfaction score and some CHW demographic characteristics, as well as factors relating to CHWs’ experiences in implementing the program. Since the CHW satisfaction score does not have any meaningful units, for the regressions, the satisfaction score was standardized (re-scaled by centering on the mean and dividing by the standard deviation (SD) in order to aid interpretation of the coefficients).

The demographic characteristics of the CHWs we assessed for associations with the satisfaction score included age, sex, education level and years of experience. Factors relating to CHW experiences in implementing the program included CHWs’ reports about community members’ attitudes towards malaria testing (whether they are aware of testing, whether they trust the test result, and whether they follow the CHWs’ advice based on the test result) and how the CHW perceives their role in the program (whether they believe that the community appreciates and recognizes their services, whether they receive enough supervision, their confidence in their abilities and whether the program has interfered with their other activities). Most of the questions on CHW experiences in the program were based on 5-point Likert scales and were dichotomized into two categories: “Strongly Agree” and all others combined (“Strongly Agree” was usually the most common response).

All analyses were conducted using Stata/IC version 12.1 [39].

## RESULTS

### Sample characteristics

Demographic data for all 70 CHWs are presented in **Table 1**. In our sample, 64% of the CHWs were female, and the mean age was 44 years (SD: 9.5 years). Forty-nine percent of CHWs were from Bungoma East sub-county and 51% from Kiminini sub-county. The majority of CHWs had completed secondary school (54%) and most of the remaining CHWs had completed primary school and had received some secondary education (34%). Twenty percent of CHWs interviewed had some post-secondary training. The majority of CHWs interviewed had served as a CHW for more than five years (54%). Most of the remaining CHWs had volunteered between three to five years (26%) or between one and three years (16%).

**Table 1:** Demographic data of all community health workers (CHWs) interviewed (N = 70)

	Count (N)	Percent (%)
**Sex:**		
Female	45	64
**Age (years):**		
20-29	3	4
30-39	18	26
40-49	29	41
50-59	19	27
60-69	1	1
**Education (highest level completed):**		
Some primary (not completed Class 8)	3	4
Primary completed	5	7
Primary completed and some secondary (secondary not completed)	24	34
Secondary completed	38	54
*Optional*		
Some college	5	7
College completed	9	13
**Years of experience as a CHW**		
<1	3	4
1-3	11	16
>3-5	18	26
>5	38	54
Roles outside of being a CHW:*		
Farmer	48	69
Business	10	14
Pastor	6	9
Shopkeeper	6	9
School board member	5	7
Volunteer with another organization	5	7
Other	21	30
None	1	1

*More than one response was allowed.

### Factors influencing motivation and satisfaction

#### Motivation

**Figure 1:**
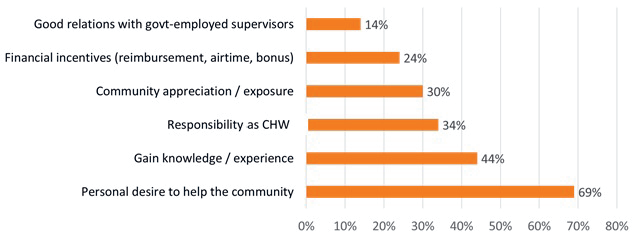
Motivators that community health workers reported for their participation in the malaria program. More than one response was allowed (N = 70).

**Figure 1** shows some of the reasons CHW commonly cited as their primary motivations for participating in the malaria diagnosis program. Most CHWs were motivated to engage in their role in this malaria program by a personal desire to help their community (69%) and many were also motivated by the knowledge and experience they gained as a result of participating (44%).

When asked about what they liked about their role in the program, CHWs said they were satisfied by the positive impact the malaria program has had on the community (29, 41%). For example, CHWs said they were able to help the community by educating community members on malaria testing, administering malaria tests, enabling quicker treatment, and providing vouchers for drugs at a subsidized rate. Many CHWs also appreciated the status and esteem they had gained as a result of their participation (17, 24%), the knowledge and skills they had gained (15, 21%), and the supervision they had received from program staff (14, 20%). All CHWs reported that they enjoyed carrying out their role in this program (68, 97% “strongly agree”; 2, 3% “somewhat agree”).

#### Challenges

Although CHWs’ overall appraisal of their role in the program was positive, they reported facing a variety of challenges in implementation. The most common challenges included low awareness among the community about malaria testing in general and the role of CHWs in malaria testing (27, 39%), problems with transportation to visit clients (21, 30%), few clients coming for testing (14, 20%), and not being able to dispense drugs directly to the client (13, 19%). Many CHWs said that increased compensation (monetary or otherwise) would help them perform their role in the malaria program better (39, 56%). CHWs also said that they would like more trainings (36, 51%) and a form of identification, such as a badge, (29, 41%).

**Figure 2:**
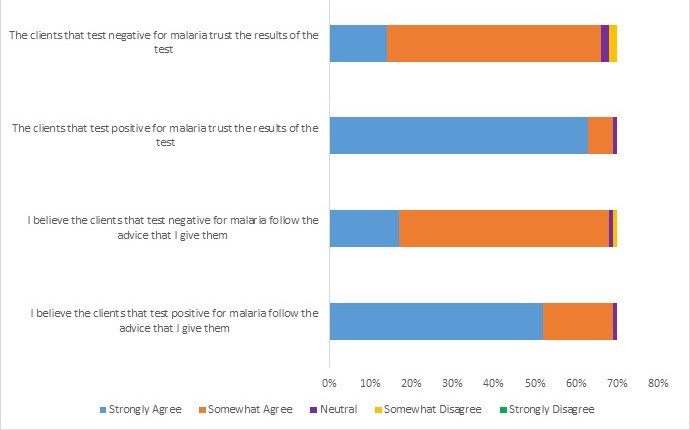
Client trust and adherence to community health workers advice for positive vs negative rapid diagnostic test (RDT) results. Only one response was allowed (N = 70).

When asked what they did not like about participating in the malaria testing program, many CHWs expressed dissatisfaction with community members’ lack of awareness about their role in the program, particularly when clients did not trust the malaria test or follow the CHWs’ health advice. CHWs believed that clients trusted positive malaria test results (63, 90% “strongly agree”) more than negative test results (14, 20% “strongly agree”). Similarly, CHWs believed that clients who tested positive for malaria followed the advice they gave those clients (52, 74% “strongly agree”) more than clients who tested negative for malaria (17, 24% “strongly agree”). (**Figure 2**) Low community awareness of malaria testing is also reflected in the fact that some CHWs agreed with the statement that clients they tested for malaria thought they were being tested for HIV (22, 31%).

Lastly, while the majority (38, 54%) of CHWs strongly agreed that the malaria RDT they perform can be trusted just as much as one performed in a health facility, there was a substantial proportion (29, 41%) of CHWs who said that they only “somewhat agreed” with the statement. It is not clear if this mistrust is directed at the test itself or reflects the CHWs’ confidence in their ability to perform the test.

### Factors related to feasibility of scale-up

**Figure 3:**
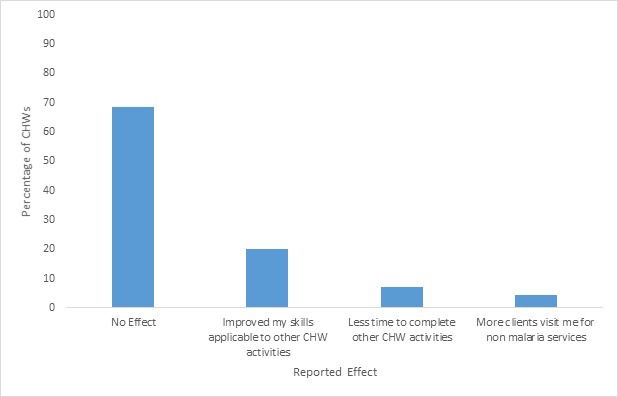
Reported effect of malaria program on community health workers’ other activities. More than one response was allowed for community health workers who answered the program has had an effect on their other CHW activities (N = 70).

The feasibility of scaling up large community-based programs depends not only CHWs’ motivation and satisfaction, but also on the degree to which the program activities either interfere with, or benefit, CHWs’ other work and non-work responsibilities. The majority of CHWs reported that their work in this malaria program had not had any effect (positive or negative) on their other CHW activities (48, 69%) (**Figure 3**). Some CHWs answered that it had improved their skills that are applicable to other CHW activities (14, 20%), though a few reported that their work with the malaria program had given them less time to complete other CHW activities (5, 7%). Some CHWs reported providing more non-malarial health services than before their involvement in malaria testing (43, 61%) and, for these CHWs, these services primarily included providing more health education (26, 61%) or maternal and/or child health services (19, 44%).

Although many CHWs said that the total time they spend on all their CHW activities since they began working with the malaria program had increased (42, 60%), almost all (69, 99%) CHWs strongly agreed that they wanted to continue their role in the malaria program. Most CHWs also reported that their work with this malaria program had not changed their ability to perform their other responsibilities outside of being a CHW (54, 77%). Some said that it had interfered somewhat with their non-CHW responsibilities (16, 23%), mainly those relating to their families (13, 19%).

### Factors associated with satisfaction

The satisfaction score created using the first principal component from the polychoric PCA ranged from -1.93 to 2.60, with a mean of -0.04 and standard deviation of 1.09 (Figure S1 in **Online Supplementary Document(Online Supplementary Document)**). The first principal component explained 33% of the variance. **Table 2** shows the association between the re-scaled satisfaction score and CHW demographic characteristics, CHWs’ reports of client awareness and trust in RDTs, and CHWs’ perceptions of their own role within the program. We find that a perceived sense of community appreciation for their work was associated with a 0.76 standard deviation increase in the satisfaction score (95% confidence interval (CI) [0.10, 1.24], *P* = 0.03). We also find some evidence that the CHW satisfaction score was associated with CHWs’ strong agreement that clients follow their advice following a negative RDT result (β = 0.54, 95% CI [-0.01, 1.08], *P* = 0.05), and CHWs’ strong agreement that their RDT can be trusted as much as an RDT done at a health facility (β = 0.41, 95% CI [-0.07, 0.88], *P* = 0.09). We found no evidence of association between the satisfaction score and age of the CHW (**Table 2**).

**Table 2:** Association between Standardized Satisfaction Score and Community Health Worker (CHW) demographics, community attitudes towards testing and CHW self-perception of role*

	Coefficient	Standard Error	*P*-value
**CHW demographic:**			
Age	0.01	0.01	0.25
Sex (female)	-0.17	0.25	0.49
Years of CHW experience	0.22	0.13	0.11
Completed secondary education	0.33	0.24	0.17
**RDT awareness and trust:**			
CHW reports everyone is aware of RDT availability	-0.28	0.26	0.28
CHW reports clients sometimes refuse RDT (vs clients never refuse RDT)	-0.42	0.31	0.17
CHW reports clients follow CHW advice for negative RDT result (Strongly Agree v. Otherwise)	0.54	0.27	0.05
CHW reports clients follow CHW advice for positive RDT result (Strongly Agree v. Otherwise)	0.04	0.28	0.89
**CHW role perceptions:**			
CHW strongly agrees he/she is appreciated by community for role in malaria project	0.76	0.33	0.03
CHW strongly agrees he/she gains recognition from role in malaria project	0.1	0.34	0.78
CHW reports any interference of time taken by malaria project work with other responsibilities	0.11	0.29	0.69
CHW strongly agrees he/she receives enough supervision on malaria project role	-0.44	0.43	0.31
CHW strongly agrees he/she is confident in performing their role in the malaria project	0.27	0.59	0.65
CHW trusts his/her RDT as much as health facility RDT	0.41	0.24	0.09

CHW – community health worker, RDT – rapid diagnostic test

*Results from uni-variate linear regressions with standardized satisfaction score (first principal component from polychoric PCA) as an outcome.

## DISCUSSION

Community Case Management (CCM) programs are being implemented and scaled-up around the world. Previous research has shown that CHW motivation and satisfaction are associated with improved performance and retention of CHWs [2,12-17]. This suggests that taking these factors into account in program design is crucial both for the well-being of CHWs as well as for the long-term success of these health programs. In this study, we measure CHW satisfaction with their role in a malaria CCM program and relate it to specific motivating factors and challenges in order to better understand the types of interventions that can promote CHW satisfaction.

Overall, CHWs in this study reported satisfaction with their role in the malaria program, particularly in terms of the positive impact it has had on the community. CHWs were motivated by an altruistic desire to help their community, consistent with evidence from previous studies that suggest altruism is a primary motivator for CHWs in their work [18-25,40,41]. The fact that the CHWs are largely intrinsically motivated is encouraging for the sustainability of the CCM programs where CHWs provide basic health care in the community. CHWs also placed a high value on gaining new skills and knowledge, suggesting that program staff may want to consider how to integrate ongoing CHW learning opportunities into programs.

CHWs reported being dissatisfied primarily when community members were not knowledgeable about malaria testing or about CHWs’ work in the malaria program. Rapid diagnostic tests (RDTs) are still a relatively new technology and are typically performed in health care facilities in the study area, rather than by CHWs. Therefore, the community members may have not yet become accustomed to both the RDTs themselves and to CHWs’ role in performing them. We found a positive association between CHW overall satisfaction and their perceptions that the community appreciated their work. Similar studies have found that lack of community acceptance and understanding of CHW programs demotivates CHWs [17,20,23,27]. This suggests that community sensitization and mobilization are important CCM program activities not just for increasing uptake of CHW services among the population, but also for boosting CHW morale. For example, working with community leaders and holding community meetings could help raise the profile of CHWs as well as clarify their roles in the community and which services they do and do not provide.

Previous studies have examined CHW compliance to positive vs negative malaria test results and have suggested that clients’ expectations may contribute to lower compliance with negative test results compared to positive ones [4,5]. However, to our knowledge, no previous research has directly assessed CHWs’ perception of clients’ acceptance of positive vs negative results. In our study, CHWs reported that clients are less likely to trust the result, or follow the advice of the CHW, when they test negative by RDT than when they test positive. We also find some evidence that CHWs who strongly agreed that their clients followed CHW advice following a negative RDT result had higher overall satisfaction scores. Clients may be less accepting of negative test results because, when a client requests an RDT test from a CHW, they may already believe that they have malaria. In order to improve the credibility of RDT results performed by CHWs, it is important for health facility staff to support CHWs efforts in providing health education and health services, including addressing any discordant results between CHW and health facility with a respectful approach. It may also help to further educate CHWs on how to discuss the meaning of negative RDT results with clients and on how to refer clients to the health facility if they test negative. This could increase CHW credibility in the community, enhance community acceptance of these negative test results, and potentially increase CHW satisfaction as well.

Additionally, several CHWs have reported in supervision meetings that there were clients who, after testing negative on the RDT performed by the CHW, subsequently visited a health facility and received a positive malaria diagnosis. While these discrepancies were infrequent, this could have an impact on CHW confidence in their testing abilities or in the reliability of the test and may undermine CHW satisfaction. Therefore, ensuring test quality, CHW confidence in test results, and support from local health facilities for CHWs’ testing activities could not only raise community trust in malaria testing but may also improve CHW satisfaction.

Though CHWs were not directly asked about the incentive structure of this malaria program, many reported that increased compensation (monetary or otherwise) would help them perform their role better. Several studies in similar settings have also reported the importance of monetary and other material incentives in motivating CHWs [13,18,19,21-25,42-44]. Therefore, CCM programs should consider both financial and non-financial incentives as potential sources of motivation for CHWs.

Overall, we find that CHWs’ primary motivations for participation, and their positive perceptions of the program, tended to be individual level factors, whereas the challenges and negative aspects of participation were generally community and organizational level factors. For example, the three main reported motivators for participating in the malaria program – a desire to help their community, the opportunity to gain knowledge and experience, and a sense of responsibility to participate because of their role as a CHW – all reflect at the level of the individual CHW. In contrast, the top three challenges mentioned by CHWs were predominantly at the community and organizational levels (frustration with a lack of understanding among clients about the study and malaria testing in general, lack of community participation, and transportation). This distinction between the influence of individual factors vs community and organizational factors on positive and negative measures of CHW satisfaction has implications for the types of interventions that may be most successful in achieving high overall satisfaction.

Although we found no evidence that CHW age was associated with the satisfaction score, we did find some suggestive evidence that additional years of experience as a CHW was positively associated with higher satisfaction. CHWs with more years of experience may have fewer frustrations than less experienced CHWs. While there may be an element of self-selection in this association (ie, those who were dissatisfied with their CHW work would probably not stay long in the role), this nonetheless indicates that veteran CHWs may be an important resource for CCM programs to draw on.

A key question in scaling up CCM programs is how the additional services provided by the CHW influences their workload and affects their other CHW and non-CHW activities. The majority of CHWs in this study reported that their work with the malaria program did not have an effect on their other CHW activities and had not changed their ability to perform their other responsibilities outside of being a CHW. Among those who did report that the program had affected their other CHW activities, most reported that the effect was positive in that it improved their skills applicable to other CHW activities. While this self-reported data is encouraging, we are unable to conclude whether the CHWs’ work with the malaria project had an actual effect on their other activities without objective measures of the time distribution of their work before and during the malaria program.

There are some limitations to this study. First, although we used independent interviewers to minimize CHWs’ inclination to provide socially desirable responses, CHWs may have been reluctant to express dissatisfaction if they suspected that the interviewers were associated with the program, or simply to avoid appearing discontented. Second, some of the open-ended survey questions included response options categorized prior to administering the survey. We kept these response options simple to make them easy for the interviewers to understand and fill out while administering the survey. However, some of these answer options could be open to multiple interpretations. For example, the majority of CHWs responded that their most common challenge in implementing the malaria CCM program was the “level of understanding of the community member.” This could refer to the community member not understanding the role of the CHW in the program, how the rapid diagnostic tests work, the purpose of the test, or other potential sources of misunderstanding.

Third, although CHWs participating in this study generally had more than three years of experience working on health issues in their communities, they did not have previous experience with community case management of acute illnesses outside of recognition and referral. Therefore, the CHWs diagnostic role was relatively new within the community. Furthermore, the CHWs were engaged in a research program testing a new intervention, which generally includes more intensive supervision than standard programmatic implementation of CCM, and this may have affected CHWs’ experiences as well as their responses.

Fourth, although this was a relatively large program, covering approximately 16 000 households, CHWs could face additional challenges in broader iCCM programs where they may have higher patient loads, lower levels of supervision and compensation, and greater likelihood of lacking one or more supplies. These challenges might reduce CHWs’ satisfaction and motivation as well as the quality of services that they provide. On the other hand, our results suggest that a broader program providing CHWs the opportunity to diagnose more conditions and provide drugs (not a component of this study) might increase CHW satisfaction.

Finally, although we find CHW satisfaction was associated with factors such as community appreciation of their work, and both CHWs’ own trust and their clients’ trust in their test, we cannot say whether these relationships are causal. In addition, our analysis of these associations may have been under-powered due to our relatively small sample size.

Nonetheless, our results indicate that, overall, CHWs are satisfied with their participation in this program and are motivated by the positive impact they are having in the community. Furthermore, their work with the program is likely contributing to the development of their skills and their other CHW work. These findings suggest that scaling-up such a program would be both feasible and sustainable though CCM programs may need to make greater efforts to address community awareness and understanding of the services that CHWs provide as well as CHWs’ desire for increased compensation. Moreover, additional research is needed to determine whether CHWs would continue to be satisfied, motivated, and provide quality care when faced with the additional organizational challenges of larger-scale CCM programs.
