# Supplementary material for: Motivation and satisfaction among community health workers administering rapid diagnostic tests for malaria in Western Kenya
Source: J Glob Health. 2018 Feb 22;8(1):010401. doi: 10.7189/jogh.08.010401 (PMC5823030; doi:10.7189/jogh.08.010401)
Supplement: Online Supplementary Document [file jogh-08-010401-s001.pdf]

Online Supplementary Document

Winn et al. Motivation and satisfaction among community health workers administering rapid diagnostic tests for malaria in Western Kenya

J Glob Health 2018;8:010401

Appendix S1.

Coupon R01 CHV Participation Assessment

May 20, 2016

Note on administration: This survey is intended to be administered orally by an enumerator to a CHV.

I. Background Information

1) CHV Code and DekiReader (*to be filled by enumerator)*:

Issued DekiReader?	
Yes	No

2) Name: ___________________ _____________________

3) Highest school level:

Circle one of the five options below:

1. None
2. Some Primary (not completed Class 8)
3. Primary completed
4. Primary completed and some secondary (secondary not completed)
5. Secondary completed

If Applicable: Circle one of the four options below:

6. Some College

7. College completed

8. Some University

9. University completed

4) For how long have you served as a CHV?
(Enumerator: If exactly 1 year or exactly 3 years, choose option 2)

1. < 1

2. 1-3 Years

3. > 3-5 Years

4. > 5 Years

5) Apart from serving as a CHV, what other occupation(s) or volunteer positions do you have?

1. Farmer

2. Shopkeeper

3. Volunteer with another organization

Write-in organization: ____________________

4. Other: ________________________________

____

6) How many trainings for CHV activities of any kind have you had this year (since January)?
(Enumerator: Read the list; Circle only one)

1. None

2. One

3. Two

4. Three
5. Four
6. Five
7. More than five

7) How long ago was your most recent training for CHV activities of any kind?
(Enumerator: Read the list; Circle only one)
   1. Within the past week
   2. Between one week and one month ago
   3. Between one and three months ago
   4. Between three months and 1 year ago
   5. More than one year ago

8) What was your most recent training about?
(Enumerator: Read the list; Circle all that apply)
   1. Malaria (this project)
   2. Malaria (another project)

      Specify: ___________________________

   3. Polio campaign
   4. Other vaccination program
   5. Maternal and child health topic not mentioned above
   6. mHealth (Mobile Health)
   7. Other: _________________________________

   ____

II. Self-description of Role

9) In the past month, what activities have you carried out as a CHV overall?
(Enumerator: Circle all that are mentioned; do not read the list; probe)

1. Malaria testing

2. Provide vouchers for malaria drugs

3. Mosquito net distribution

4. Provide other malaria-related services/counseling

5. Polio campaign participation

6. Other vaccination campaign(s)

7. Maternal and child health topic not mentioned above

8. Giving health education to households

9. None

10. Other: ________________________________

____

____

10) What do you think are the most important services you provide as a CHV?
(Enumerator: Circle all that are mentioned; do not read the list; probe)

1. Malaria testing

2. Provide vouchers for malaria drugs

3. Mosquito net distribution

4. Provide other malaria-related services/counseling

5. Polio campaign participation

6. Other vaccination campaign(s)

7. Maternal and child health services not mentioned above

9. Giving health education to households

10. None

11. Other: ________________________________

____

____

11) What do you think is the most important service you provide as a CHV?
(Enumerator: Only allow one answer; do not read the list)

1. Malaria testing
2. Provide vouchers for malaria drugs
3. Mosquito net distribution
4. Provide other malaria-related services/counseling
5. Polio campaign participation
6. Other vaccination campaign(s)
7. Maternal and child health services not mentioned above
8. Giving health education to households
9. None
10. Other: ________________________________

____

____

12) How many times have you met with your CHEW in the past 3 months?
(Enumerator: Circle only one)

1. Once

2. Twice

3. Three times

4. More than three times

5. None

13) What are the requirements for a client to receive an RDT under this malaria project?
(Enumerator: Circle all that are mentioned; do not read the list; probe)

1. No requirements – anyone who asks can be tested

2. Must live in my CU

3. Has a documented fever (i.e., as measured by me)

4. Has a documented fever or reports a history of recent fever

5. Has a documented fever or reports a history of recent fever or reports any symptoms of malaria

6. Consent must be provided by the client (or by the parent/guardian of a minor)

7. Must not be visibly pregnant

8. Other: ________________________________

____

____

14) What motivates you to participate in this malaria project?
(Enumerator: Circle all that are mentioned; do not read the list; probe)

1. Personal desire to help the community

2. Responsibility as a CHV

3. Family

4. Organization (partner and GOK)

5. Monetary incentive (money given to group after 6 months and transport to attend supervision and supplies replenishment)
6. In-kind incentive (e.g., airtime for your phone)
7. Good relations with link facility staff, including CHEWs
8. Other: ________________________________

____

III. Satisfaction with Role

15) What do you like about participating in this project?

(Open-ended response; probe): ____

____

____

____

16) What don't you like about participating in this project?

(Open-ended response; probe): ____

____

____

____

Please indicate your level of agreement with the following statements with regards to this malaria project **only.**

(Enumerator: Read the above statement then the five options: "strongly agree", "somewhat agree"… after reading each question.)

Question	Strongly Agree	Somewhat Agree	Neutral	Somewhat Disagree	Strongly Disagree
17) The people in my community appreciate my work on this malaria project.					
18) I have gained more recognition in my community since I began testing for malaria.					
19) I believe the clients that test positive for malaria follow the advice that I give them					
20) I believe the clients that test negative for malaria follow the advice that I give them					
21) I receive enough supervision in my work from the malaria project study staff.					
22) Overall, I am confident in my ability to perform my role in this malaria project.					
23) Overall, I enjoy carrying out my role in this malaria project.					
24) I would like to continue with my role in this malaria project.					

25) *If the CHV answers "50-50", somewhat disagree" or "strongly disagree" to continuing their role in the project*, why?
(Enumerator: Circle all that are mentioned; do not read the list; probe)

1. Requires too much time
2. Requires too much effort
3. Don't receive enough appreciation
4. Don't receive compensation
5. Frustrated that they can't provide drugs directly to clients
6. Conflicts with other CHV activities
7. Conflicts with non-CHV activities / responsibilities

For 7: Record activities mentioned: ______________________

8. Other: ____

____

____

IV. Logistics & Challenges of Role

A. Implementation

i. Community awareness & trust of services

26) Are people in your village aware that you provide RDT testing?
(Enumerator: Read all the options, circle only one)

1. Everyone is aware
2. Most are aware
3. Some are aware
4. Most are *not* aware
5. None are aware

27) Are the people in your village aware that you provide a voucher for malaria drugs if RDT is positive?
(Enumerator: Read all the options, circle only one)

1. Everyone is aware
2. Most are aware
3. Some are aware
4. Most are *not* aware
5. None are aware

28) How do you think your clients learn about the malaria services (testing and vouchers) that you provide as a part of this malaria project?
(Enumerator: Circle all that are mentioned; do not read the list; probe)

1. From me
2. From family members
3. From neighbors / friends
4. From village leaders
5. From health facility staff
6. From malaria project staff
7. Other: _________________________________

____

____

Please indicate your level of agreement with the following statements.

(Enumerator: Before reading the questions, explain: "For the next four questions, answer 'strongly agree', 'somewhat agree,' '50-50,' 'somewhat disagree,' or 'strongly disagree'" Then restate the five options after reading each question.)

Question	Strongly Agree	Somewhat Agree	Neutral	Somewhat Disagree	Strongly Disagree
29) The malaria RDT that I do can be trusted just as much as a malaria RDT done in a health facility.					
30) The clients that test positive for malaria trust the results of the test					
31) The clients that test negative for malaria trust the results of the test					
32) People I test for malaria believe I am actually testing for HIV.					

33) Do any of your clients refuse an RDT when you offer it?

1. Yes
2. No

34) *If Yes to above*, What are the reasons that you think these clients refuse?
(Enumerator: Circle all that are mentioned; do not read the list; probe)

1. They don't understand why it is necessary
2. They don't trust me
3. They don't trust the test
4. They are already convinced they have malaria
5. They plan to buy antimalarial drugs regardless of test result
6. They don't want to be pricked
7. They think it may really be an HIV test
8. Other: ________________________________

____

____

ii. Logistics & Challenges

35) What are all of the ways that clients contact you to receive a malaria RDT?
(Enumerator: Circle all that are mentioned; do not read the list; probe)

1. Clients contact me at my home
2. Clients contact me at a health facility
3. Clients contact me at church
4. I contact clients at their homes
5. Clients contact me at another public location
6. Clients contact me at another private location
7. Clients call my mobile phone
8. The study staff contacts me

9. The health facility contacts me

10. Other: ________________________________

____

____

36) Where do you do testing for malaria?
(Enumerator: Circle all that are mentioned; do not read the list; probe)

1. At my home

2. At a health facility

3. At church

4. At client's home

5. At another public location

6. At another private location

7. At CHV Office

8. In Barazas (public meeting)

9. Other: ________________________________

____

____

37) How often do you lack any of the supplies you need to perform the malaria services of testing and providing vouchers when necessary?
(Enumerator: Read the following five options; Circle only one)

1. I am always lacking a necessary supply

2. I sometimes have all of the supplies I need

3. I always have all of the supplies I need

38) Which, if any, supplies related to the malaria services you perform for this project have you lacked in the past month?
(Enumerator: Circle all that are mentioned; do not read the list; probe)

1. RDTs
2. Buffer
3. Lancets
4. Pipettes
5. Alcohol swabs
6. Cotton wool
7. Gloves
8. Safety disposal box
9. Yellow waste bags
10. Plastic (zip-loc) bags
11. Thermometer
12. Thermometer batteries
13. Pen
14. Encounter forms
15. Voucher forms
16. None
17. Other: _________________________________

____

____

39) What are the main challenges you face in implementing this malaria project?
(Enumerator: Circle all that are mentioned; do not read the list; probe)

1. The clients don't trust the test.

2. Transport

3. Not enough training

4. Not enough supervision from study staff and/or CHEWs.

5. Lack of supplies

6. Competing activities

7. Very few clients coming for test

8. Nature of the community not willing to interact with CHVs

9. Cultural practices e.g use of herbal medicine, seeking help from traditional healers

10. Level of understanding of community member

11. Lack of mobilization at the community

12. Family relationships are affected

13. Other: ________________________________

____

____

40) How often would you prefer supervision to be carried out?
(Enumerator: Read the list; Circle only one)

1. Once a week
2. Once every two weeks
3. Once a month
4. Once every two months
5. Other: ______________________________

41) Why do you think we supervise your work with this malaria project?
(Enumerator: Circle all that are mentioned; do not read the list; probe)

1. To monitor whether I make any mistakes
2. To provide additional training
3. To ensure data quality
4. Other: _________________________________

42) What would help you perform your role in this malaria project better?
(Enumerator: Circle all that are mentioned; do not read the list; probe)

1. More training
2. More supervision from study team
3. More supervision from CHEWs
4. More regular restocking of supplies
5. Raising community awareness about the project
6. Compensation for time spent
7. Sharing my CHV duties with another CHV
8. Other: ________________________________

____

____

iii. Time required

43) On average, how long does it take you to complete a visit with a patient complaining of malaria symptoms?
(Enumerator: Be sure to record the time in minutes)
____________________ Minutes

44) How many hours do you think you spend on all your CHV activities combined in an average week?
(Enumerator: Read the examples in parentheses; Be sure to record the time in hours; PROBE)

____________________ Hours

45) How of those total hours do you think are for this malaria project work in an average week?
(Enumerator: Read the examples in parentheses; Be sure to record the time in hours; PROBE)

____________________ Hours

46) Do you think the total time you spend on all CHV activities has decreased, increased, or stayed about the same since you began the activities for this malaria project?
(Enumerator: Read the list; Circle only one)

1. Decreased
2. Stayed about the same
3. Increased

B. Relation to other CHV activities

47) Has participating in this malaria project had an effect on your other CHV activities?

1. Yes
2. No

48) If yes, in what ways:

(Enumerator: Circle all that are mentioned; do not read the list; probe)

1. Improved skills applicable to other CHV activities
2. More clients visit me for non-malaria services
3. Less time to complete other CHV activities
4. Other: ___________________________________

49) Please indicate to what extent you agree with the statement: Due to my involvement with this malaria project, I now provide more non-malaria health services than before this malaria project.
(Enumerator: Read the following five options; Circle only one; probe)

1. I provide much more non-malaria health services than before
2. I provide somewhat more non-malaria health services than before
3. The amount of non-malaria health services I provide has stayed the same
4. I provide somewhat less non-malaria health services than before
5. I provide much less non-malaria health services than before

(Only ask the next 2 questions if 1 "much more" or 2 "somewhat more" above):

50) If 1 "much more" or 2 "somewhat more" above: What are the other (non-malaria) services you provide more of due to your involvement in this project?
(Enumerator: Circle all that are mentioned; do not read the list; probe)

1. Maternal and child health services
2. Vaccinations
3. Providing health information
4. Other: ______________________________

____

____

51) If 1 "much more" or 2 "somewhat more" above: To whom do you provide more non-malaria services than before this malaria project started? (select all that apply):
(Enumerator: Circle all that are mentioned; do not read the list; probe)

1. Men
2. Women
3. Children
4. Other: ____________________________

52) In the last month, how if at all, has the amount of time required for your CHV work affected your ability to perform your other responsibilities not related to community health work?
(Enumerator: Read the following three options; Circle only one)

1. There has been no change in my ability to perform my other responsibilities.
2. My work as a CHV has interfered somewhat with my other responsibilities
3. My work as a CHV has interfered very much with my other responsibilities.

53) What, if any, responsibilities outside of your role as a CHV have been affected?
(Enumerator: Circle all that are mentioned; do not read the list; probe)

1. Childcare
2. Family responsibilities
3. Other work
4. Other volunteering / community activities
5. Religious activities
6. None
7. Other: ________________________________

____

____

<u>If Deki Reader circled "yes" in question 1 on the front page, ask the following three questions (64-66)</u>
<u>If "No," skip these questions</u>

54.) To what extent would you agree with the statement, "The DekiReader was easy to use correctly"?
(Enumerator: Read the following five options; Circle only one)

1. Strongly agree
2. Somewhat agree
3. 50-50
4. Somewhat disagree
5. Strongly disagree

55.) To what extent would you agree with the statement, "The Deki Reader helped me improve my ability to perform RDTs correctly"?
(Enumerator: Read the following five options; Circle only one)

1. Strongly agree
2. Somewhat agree
3. 50-50
4. Somewhat disagree
5. Strongly disagree

56.) If you had the option to use the Deki Reader for every test as part of your routine work, would you want to have it or not?
(Enumerator: Circle only one)

1. Yes
2. No
3. Don't Know / Unsure

★ ***<u>CONTINUE TO THE FINAL PAGE TO ASK THE LAST QUESTION (57)!</u>*** ★

57.) Do you have any concerns or suggestions to improve the implementation of this malaria project?
(Open-ended response; probe):
____

____

____

____

____

____

____

Thank you for your participation and feedback!

Figure S1

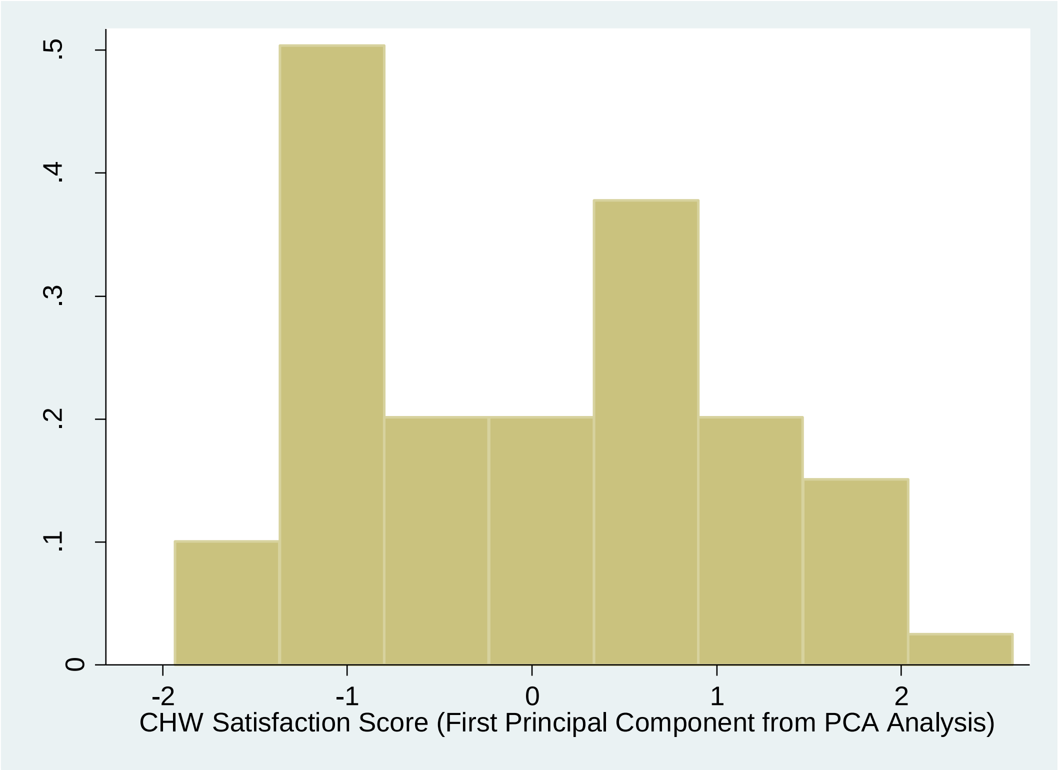

Appendix Figure A1. Distribution of CHW satisfaction score (First Principal Component from principal components analysis)
